# Decoding Glioblastoma Heterogeneity: Neuroimaging Meets Machine Learning

**DOI:** 10.1227/neu.0000000000003260

**Published:** 2024-11-21

**Authors:** Jawad Fares, Yizhou Wan, Roxanne Mayrand, Yonghao Li, Richard Mair, Stephen J. Price

**Affiliations:** * Department of Clinical Neurosciences, Academic Neurosurgery Division, University of Cambridge, Cambridge, UK;; ‡ Cambridge Brain Tumour Imaging Laboratory, Department of Clinical Neurosciences, Academic Neurosurgery Division, University of Cambridge, Cambridge, UK;; § Department of Neurological Surgery, Feinberg School of Medicine, Northwestern University, Chicago, Illinois, USA

**Keywords:** IDH-wildtype glioblastoma, Neuroimaging, Magnetic resonance imaging, Diffusion tensor imaging, Artificial intelligence

## Abstract

Recent advancements in neuroimaging and machine learning have significantly improved our ability to diagnose and categorize isocitrate dehydrogenase (IDH)-wildtype glioblastoma, a disease characterized by notable tumoral heterogeneity, which is crucial for effective treatment. Neuroimaging techniques, such as diffusion tensor imaging and magnetic resonance radiomics, provide noninvasive insights into tumor infiltration patterns and metabolic profiles, aiding in accurate diagnosis and prognostication. Machine learning algorithms further enhance glioblastoma characterization by identifying distinct imaging patterns and features, facilitating precise diagnoses and treatment planning. Integration of these technologies allows for the development of image-based biomarkers, potentially reducing the need for invasive biopsy procedures and enabling personalized therapy targeting specific pro-tumoral signaling pathways and resistance mechanisms. Although significant progress has been made, ongoing innovation is essential to address remaining challenges and further improve these methodologies. Future directions should focus on refining machine learning models, integrating emerging imaging techniques, and elucidating the complex interplay between imaging features and underlying molecular processes. This review highlights the pivotal role of neuroimaging and machine learning in glioblastoma research, offering invaluable noninvasive tools for diagnosis, prognosis prediction, and treatment planning, ultimately improving patient outcomes. These advances in the field promise to usher in a new era in the understanding and classification of IDH-wildtype glioblastoma.

**ABBREVIATIONS:**
ADC: apparent diffusion coefficient
CNNs: Convolutional Neural Networks
MD: mean diffusivity
rCBV: relative cerebral blood volume
SVZ: subventricular zone.

Isocitrate dehydrogenase (IDH)-wildtype glioblastoma is an aggressive brain tumor, characterized by heterogenous tumoral behavior, therapeutic responses, and survival outcomes.^1^ Identifying preoperative prognostic markers for stratifying patients with IDH-wildtype glioblastoma is crucial for optimizing therapeutic strategies, designing personalized therapies, and improving overall patient outcomes.^2^

Neuroimaging is crucial in glioblastoma care, aiding in screening, diagnosis, staging, treatment response evaluation, and disease surveillance. When histological diagnosis is difficult, advanced imaging such as MRI and diffusion tensor imaging (DTI) is essential. T1-weighted MRI highlights active glioblastoma regions, often corresponding to the tumor core. However, infiltration typically extends beyond the areas shown on T1-weighted and T2/fluid-attenuated inversion recovery (FLAIR) images, which assess edema and diffuse involvement.^3-5^ Contrast enhancement correlates with neovascularity, blood-brain barrier disruption, and tumor infiltration,^6^ often indicating malignancy.^7,8^

Neuroimaging further guides neurosurgeons in planning and performing surgeries, optimizing the extent of resection, and minimizing damage to vital brain regions. DTI, an advanced MRI sequence, detects microstructural changes by assessing water diffusion using key metrics—mean diffusivity (MD) and fractional anisotropy (FA).^9-11^ MD is sensitive to cellular swelling, aiding in early stroke detection,^12^ while FA measures anisotropy, reflecting white matter integrity.^13^ Previous studies have shown these metrics can predict glioblastoma survival.^14,15^

Radiomics transforms neuroimages into mineable data, extracting quantitative features related to shape, intensity, texture, and spatial relationships. This noninvasive approach characterizes tumors, aiding in predicting treatment outcomes, assessing disease characteristics, revealing biological pathways, and informing clinical decisions.^16,17^ In certain scenarios, radiomic features surpass clinicopathological factors in prognostic determination,^18-20^ serving as valuable tumor biomarkers for improved classification of IDH-wildtype glioblastoma.^21^

Machine learning techniques such as Convolutional Neural Networks (CNNs), Support Vector Machines, and Random Forests have revolutionized radiomics analysis in glioblastoma research.^22^ These methods aid in detecting genetic mutations,^23^ handling missing data,^24^ reducing artifacts,^25^ and removing noise.^26,27^ CNNs are particularly effective for tasks such as segmentation, noninvasive genomic biomarker identification,^28,29^ progression detection, and survival prediction.^30^ Machine learning models have shown strong performance with high accuracy, sensitivity, specificity, and precision.^22,31,32^

Neuroimaging increasingly uses machine learning and radiomics for tasks such as predicting patient outcomes, semi-automated tumor delineation, and diagnosis. This review highlights how recent advances enhance glioblastoma classification, identify invasive tendencies, and predict survival. Understanding glioblastoma's intertumoral heterogeneity is crucial for designing clinical trials and targeted therapies. However, the discussed imaging techniques and biomarkers are still research-based and not widely used in current clinical practice, offering a glimpse into future potential for neuro-oncology. Many of these imaging modalities, though well-established in clinical practice, are technically considered off-label uses.

## POTENTIAL RADIOLOGICAL MARKERS OF TUMORAL BEHAVIOR

### Glioblastoma Tumor Components

**FIGURE 1.**
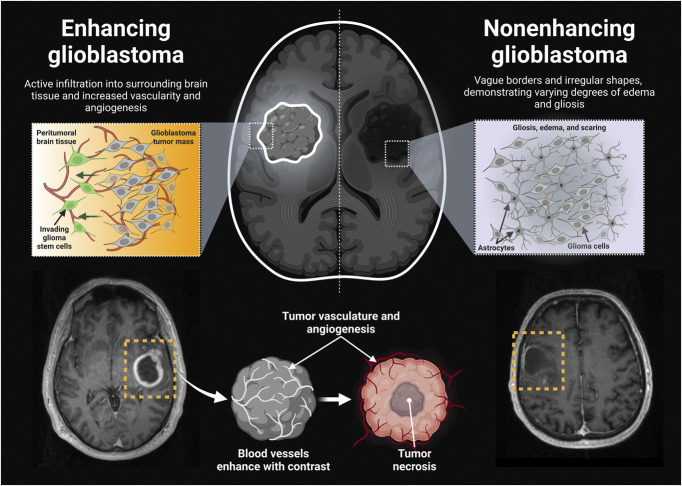
Enhancing vs nonenhancing glioblastoma. Enhancing glioblastomas typically exhibit bright contrast enhancement on imaging, indicating actively growing neoplastic tissue with increased vascularity and angiogenesis. Nonenhancing glioblastomas, on the other hand, pose challenges in delineation and quantification, often manifesting as diffuse T2 signal abnormalities associated with edema, treatment effects, and gliosis.

Glioblastomas consist of enhancing and nonenhancing tumor components, each with distinct characteristics and implications for diagnosis, treatment, and patient management. Enhancing tumor regions reflect actively growing neoplastic tissue and are assessed based on contrast accumulation^33,34^ (Figure 1). The breakdown of the blood-brain barrier leads to increased vascularity and angiogenesis in these areas.^35^ Evaluation of treatment response primarily focuses on changes in enhancing tumor size, but antiangiogenic therapies have posed challenges due to reduced contrast enhancement.^36,37^ Nonenhancing tumor regions lack contrast uptake and present difficulties in accurate delineation and quantification.^38^ They exhibit T2-signal abnormalities associated with edema, treatment effects, and gliosis. Additional imaging techniques such as FLAIR are used to improve detection of nonenhancing tumor components.^35^ The distinction between enhancing and nonenhancing tumor components is critical for surgical resection, radiation therapy, and treatment monitoring. Although quantifying nonenhancing tumors remains challenging, efforts are underway to develop physiological imaging techniques for obtaining quantitative data on tumor burden.

### Glioblastoma Proximity to Ventricles

**FIGURE 2.**
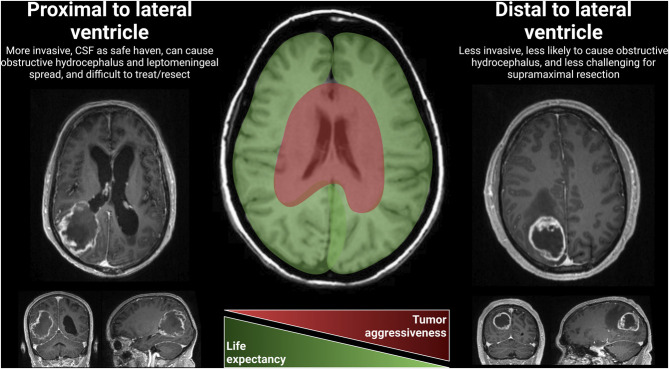
Proximity to lateral ventricles. Close to ventricles: unique growth patterns and invasive behavior due to the anatomical and molecular properties of the ventricular region. Surgical resection becomes more challenging when the tumor involves or is adjacent to the ventricles, and adjuvant therapies may be less effective due to the presence of CSF as a sanctuary site for residual tumor cells. Obstructive hydrocephalus and leptomeningeal metastases are common complications. CSF, cerebrospinal fluid.

Glioblastomas that are near the ventricles exhibit distinct characteristics and have a significant impact on clinical outcomes and treatment strategies (Figure 2). These glioblastomas often show unique growth patterns and invasive behavior due to the anatomical and molecular properties of the ventricular region.^39^ Surgical resection becomes more challenging when the tumor involves or is adjacent to the ventricles, and adjuvant therapies may be less effective due to the presence of cerebrospinal fluid as a sanctuary site for residual tumor cells.^40^ Glioblastomas near the ventricles are associated with increased aggressiveness, higher rates of tumor recurrence, and decreased overall survival.^41^ Obstructive hydrocephalus and leptomeningeal metastases are common complications.^42^ Tailored approaches to clinical management are necessary, including advanced imaging techniques for preoperative evaluation, careful adjustment of adjuvant therapies, and close monitoring for complications. In a prospective study, the outcomes and MRI characteristics of ventricle-contacting and non-contacting glioblastoma were compared. Patients with ventricle-contacting tumors had lower survival rates, and perfusion values in the peritumoral region were higher, indicating a more aggressive behavior.^43^ Another study used DTI metrics, such as FA and MD, to investigate the involvement of the subventricular zone (SVZ) in glioblastoma. The SVZ with high FLAIR signal showed increased isotropic values and lower anisotropic values, suggesting tumor infiltration and the presence of therapy-resistant stem cells.^44^ SVZ gliomas were associated with shorter overall survival and progression-free survival but did not exhibit a different immunohistochemical pattern for specific markers.^44^

### Pseudoprogression vs Tumor Progression

The emergence of diverse therapies, including immunotherapy, has led to complex inflammatory responses, complicating the differentiation between pseudoprogression (a radiation-induced treatment effect) and true tumor progression in glioblastoma management. A hybrid machine learning algorithm combining CNNs and long short-term memory networks was used to differentiate between pseudoprogression and true tumor progression in glioblastoma.^45^ The CNNs analyzed 9 axial postcontrast T1-weighted MRI images. This number ensures practical model training without excessive resource demands. By identifying patterns and characteristics within the MRI data, the CNNs helped distinguish between pseudoprogression and true progression based on spatial information.^45^ The long short-term memory component then integrated the CNN-derived features with clinical data to further improve the diagnostic accuracy. The model achieved an area under the curve of 0.83, demonstrating its effectiveness in distinguishing between pseudoprogression and true progression.^45^

3D shape features of glioblastoma on MRI can further help in differentiating between pseudoprogression and tumor progression in patients with glioblastoma. In a multicenter study, using the enhancing lesion on T1-weighted imaging and T2-weighted/FLAIR hyperintensities enhanced the accuracy of distinguishing these radiographically similar pathologies on routine multiparametric MRI scans.^46^ The top discriminative features, capturing total curvature of the enhancing lesion and curvedness of the perilesional region, achieved an accuracy of 90.2% in distinguishing these radiographically similar pathologies, suggesting potential for improved differentiation beyond traditional bidirectional measurements.^46^ Longitudinal DTI data can also distinguish pseudoprogression from tumor progression. Using discriminative dictionary learning methods to extract distinguishing features and support vector machines for classification achieved a high average accuracy of 0.867 and an area under the curve of 0.92.^47^ Thorough sensitivity analysis was conducted to optimize overall performance.^47^

### DTI and Connectome Disruptions

**FIGURE 3.**
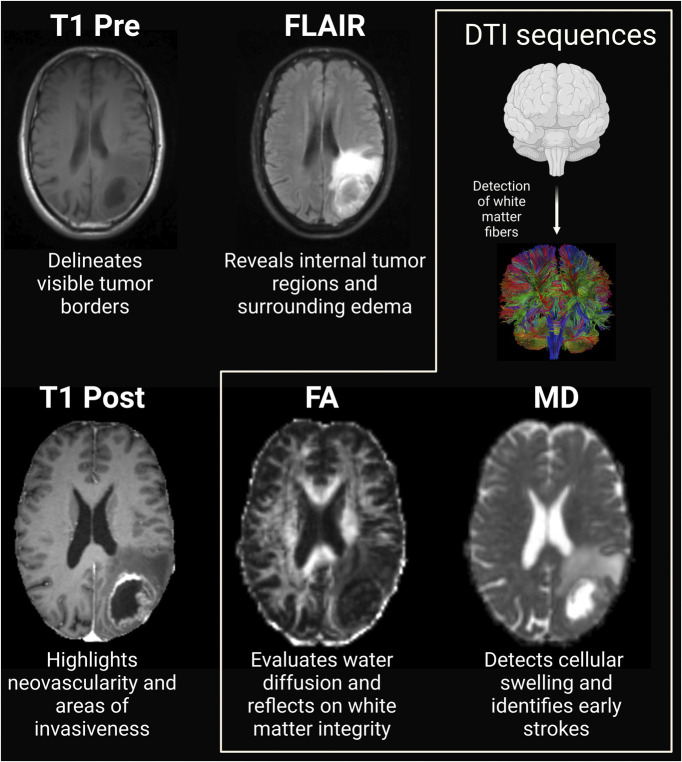
MRI and DTI sequences for glioblastoma characterization. T1 Pre delineates visible tumor borders, FLAIR and T2 highlight internal tumor regions and edema. Contrast enhancement on MRI indicates neovascularity and infiltration. DTI assesses tissue microstructure through water diffusion, evaluating white matter tract integrity using MD and FA. MD detects cellular swelling, aiding early stroke detection, while FA measures water diffusion orientation, reflecting white matter integrity. DTI, diffusion tensor imaging; FA, fractional anisotropy; FLAIR, fluid-attenuated inversion recovery; MD, mean diffusivity.

DTI detects tumor infiltration by reflecting microstructural disruption (Figure 3). In a study of 115 patients with glioblastoma, decreased isotropic diffusion and increased anisotropic diffusion in nonenhancing regions indicated a more infiltrative tumor habitat and potential treatment target.^14^ Decreased isotropic diffusion suggests higher cell density, correlating with more aggressive tumor areas, while increased anisotropic diffusion reflects preserved white matter fibers that may aid tumor migration. These diffusion patterns (p↓/q↑) are linked to patient survival and tumor progression, highlighting their potential as targets for focused radiotherapy.^14^

**FIGURE 4.**
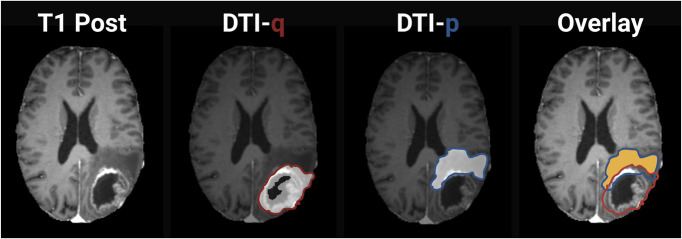
DTI-derived measures: p and q representing diffusion properties. p (isotropic, blue) indicates magnitude; q (anisotropic, red) reflects directionality. Yellow region signifies infiltrating tumor margin. DTI, diffusion tensor imaging.

Quantification of the brain structural connectome can also predict glioblastoma invasion. Using 2 independent prospective glioblastoma cohorts (a test/training cohort and a validation cohort) of 117 and 42 patients, respectively, white matter connection pathways were constructed between brain regions using DTI of healthy subjects.^48^ The connectome disruptions in patients with glioblastoma were widespread in the normal-appearing brain beyond focal lesions and were associated with lower preoperative performance, impaired cognitive function, and worse survival.^48^ In a cohort of 49 patients, DTI imaging and coregistration of preoperative and recurrence sequences revealed that the abnormal anisotropic component (q) of DTI had significantly higher sensitivity than the contrast-enhancing region for predicting glioblastoma recurrence^49^ (Figure 4). Combining the abnormal q region with the contrast-enhancing region resulted in even higher sensitivity without compromising specificity,^49^ suggesting that these imaging biomarkers may enhance recurrence prediction. In addition, the q component of DTI metrics was correlated with overall survival.^49^ In a separate cohort of 33 patients with glioblastoma, median voxel q values of the whole brain, contrast-enhancing hemisphere, and non–contrast-enhancing hemisphere significantly correlated with overall survival,^50^ highlighting the potential of q component as a prognostic biomarker.

### Radiological Characterization of Metabolic Heterogeneity

The use of imaging with labeled radiotracers enables the evaluation of metabolic heterogeneity in glioblastoma, which result from genomic alterations and microenvironmental factors that cause metabolic reprogramming.^51^ The metabolic identity of the tumor determines its sensitivity or resistance to chemoradiotherapy.^52-54^ Glycolysis is generally downregulated in highly proliferative glioblastoma cells and stem cells, which contributes to radiation resistance.^55^ Neuroimaging with hyperpolarized carbon 13–labeled metabolites assesses tumor metabolism, crucial for understanding the aggressiveness of glioblastoma. The lactate-to-pyruvate ratio reveals metabolic activity, with higher ratios indicating increased glycolysis, a hallmark of aggressive tumors due to the Warburg effect. Elevated lactate dehydrogenase A levels, correlating with this ratio, reflect enhanced glycolytic flux and poor prognosis.^56^ The study confirmed a link between the lactate-to-pyruvate ratio and lactate dehydrogenase A expression,^57^ highlighting the connection between metabolic activity and glioblastoma aggressiveness.

## ROLE OF RADIOMICS IN GLIOBLASTOMA PROFILING

Radiomics allows the extraction and analysis of quantitative features from neuroimaging. By leveraging advanced imaging techniques and computational methods, radiomics provide valuable insights into the characteristics, behavior, and prognosis of glioblastoma.

### Glioblastoma Profiling Based on Radiomics

Glioblastoma tumors, known for their invasiveness and heterogeneity, can be differentiated based on imaging biomarkers such as apparent diffusion coefficient (ADC) and relative cerebral blood volume (rCBV), which have implications for patient survival outcomes. Regions of low ADC and high rCBV, present in both contrast-enhancing and nonenhancing tumor regions, were associated with poor survival outcomes.^58-61^ Similarly, another study focused on low perfusion compartments in glioblastoma and identified 2 compartments based on overlapping regions of ADC and rCBV quartiles: high ADC-low rCBV and low ADC-low rCBV.^62^ These compartments exhibited higher lactate and macromolecule/lipid levels compared with normal white matter. The size of the low ADC-low rCBV compartment, which correlated with greater tumor infiltration, was found to be a significant predictor of survival outcomes. Patients with higher lactate levels in the low ADC-low rCBV compartment had worse overall survival.^62^ This suggests that the low ADC-low rCBV compartment may serve as a measurable indicator of treatment-resistant regions in glioblastoma, contributing to poor prognosis.

Epidermal growth factor receptor variant III, a driver mutation and therapeutic target in glioblastoma, necessitates precise identification for effective treatment stratification. A noninvasive multiparametric MRI method integrating diverse imaging features, including spatial distribution patterns, yielded an 85.3% cross-validated accuracy in an independent cohort and 87% in a replication cohort.^63^ EGFRvIII+ tumors displayed elevated rCBV, reduced ADC, increased FA, and diminished T2-FLAIR signal. These characteristics are indicative of neovascularization, inflammation, and necrosis. In addition, EGFRvIII+ tumors exhibited a heterogeneous spatial pattern within the frontal and parietal lobes, while EGFRvIII- tumors were predominantly located in the temporal lobe.^63,64^ This approach provides a comprehensive evaluation of tumor spatial heterogeneity, offering potential preoperative patient stratification for EGFRvIII-targeted therapies.^63^

Moreover, integrating histogram features from multiparametric MRI provided valuable insights into survival outcomes in glioblastoma. A multiview approach using histogram features identified 2 distinct patient clusters.^65^ The cluster characterized by lower N-acetylaspartate/creatine ratio in the nonenhancing region exhibited better overall survival and progression-free survival.^65^ Furthermore, higher mean anisotropic diffusion in the nonenhancing region was associated with worse survival outcomes.^65^ These findings highlight the potential of selected histogram features as prognostic markers in glioblastoma, offering additional information for predicting patient survival at the 12-month mark.

### Integration of Machine Learning in Radiomics for Diagnosing IDH-Wildtype Glioblastoma

**FIGURE 5.**
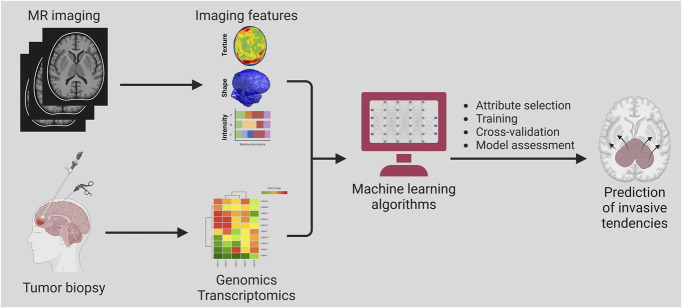
Role of machine learning in subtype prediction in glioblastoma. Training a residual Convolutional Neural Network on preoperative MRI and/or histology samples of gliomas allows the noninvasive diagnosis and molecular profiling of glioblastoma and the prediction of prognosis and survival outcomes. MR, magnetic resonance.

**FIGURE 6.**
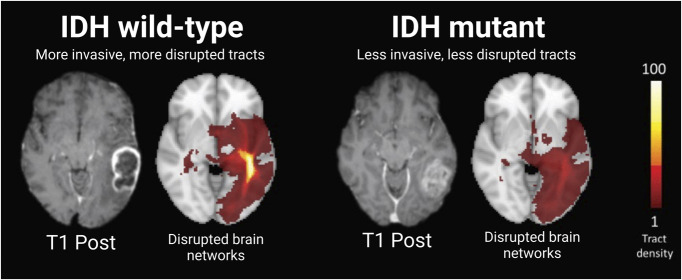
Voxel distribution of disrupted brain networks. Visualization of the brain networks in IDH wild-type gliomas show a higher density of disrupted brain networks and tracts compared with IDH-mutant gliomas, consistent with the understanding that IDH wild-type tumors are generally more invasive.^73^

Integration of machine learning in radiomics offers a noninvasive method that may aid in the diagnosis and molecular characterization of glioblastoma. Training a residual CNN on preoperative magnetic resonance imaging of gliomas allows the prediction of IDH status.^66^ Several studies testing these tools achieved 79% to 94% prediction accuracy of IDH status in patients with high-grade gliomas.^66-68^ Machine learning models using structural brain networks and graph neural networks can be powerful tools for predicting IDH mutation status based on pretreatment MRI. The systemic brain alterations caused by glioblastoma invasion along white matter tracts can be characterized using these networks,^69^ facilitating the prediction of IDH mutation with high accuracy, sensitivity, and specificity rates of 87%, 88%, and 86%, respectively.^70^ Another deep learning neural network developed to identify relevant genetic mutations in glioblastoma could determine IDH1 mutation status with 94% accuracy, 1p/19q codeletion with 92% accuracy, and 06-methylguanine-DNA methyltransferase promoter methylation status with 83% accuracy.^71^ Further integrating glioblastoma geometrics into convolutional and graph neural networks can improve IDH mutation prediction and glioblastoma characterization^72^ (Figure 5). A multimodal learning framework combining focal tumor imaging, geometric features, and brain network data from MRI improved IDH mutation prediction, outperforming existing deep learning models (Figure 6).^73^ In addition, integrating radiomics with machine learning models can efficiently classify glioblastoma transcriptome subtypes. An XGBoost-based radiomics model used handcrafted radiomics features that include volumetric measures (nonenhancing tumor volume relative to peritumoral edema), textural features from FLAIR volumes (entropy, size zone matrix, run-length matrix, and neighborhood gray-tone difference matrix parameters), and tumor growth model parameters (estimated growth time).^74^ The model achieved acceptable predictive accuracies for the classical (70.9%), mesenchymal (73.3%), neural (88.4%), and proneural (88.4%) subtypes of high-grade gliomas.^74^

### Integration of Machine Learning in Radiomics for Predicting Tumor Behavior and Survival

**FIGURE 7.**
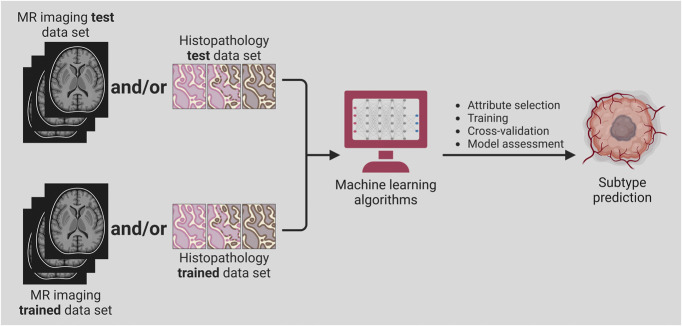
Role of machine learning in predicting glioblastoma behavior. The use of machine learning to incorporate radiomics and genomics provides a novel approach that could assist in determining the invasive tendencies of glioblastoma. MR, magnetic resonance.

Radiomics, particularly using CNNs on MRI, holds promise in predicting glioblastoma tumor behavior and patient survival, particularly when combined with clinical variables.^75^ By delineating areas of high local progression beyond the contrast-enhancing rim using preoperative MRI, radiomics aids in optimizing treatment strategies (Figure 7). Using CNNs, it has been demonstrated that peritumoral progression areas exhibit distinct imaging characteristics, including higher signal intensity in FLAIR, rCBV, and T1 contrast, and lower intensity in ADC and DTI-p.^76^ These findings suggest the potential of radiomics to enhance clinical management, with reported accuracies ranging from 78% to 93%.^76^ Volumetric features extracted through CNNs from preoperative MRI also correlate with survival, with ratios of contrast-enhancing and necrotic core regions to the tumor core independently associated with overall survival.^77^ Furthermore, with 90.6% accuracy, analysis of structural and functional MRI data using a CNN can predict survival categories (<1 year, 1-2 years, >2 years) of patients with glioblastoma pretreatment.^78^ Cortical thickness measurements in the superior temporal sulcus and parahippocampal gyrus and connectivity in the dorsal and inferior somatomotor networks, visual networks, and cingulo-opercular networks played a crucial role in the predictive model.^78^ Integrating positron emission tomography (PET) imaging with MRI-based radiomics by using various tracers could further refine these predictions by adding metabolic and molecular insights into the analysis. For instance, PET radiomics has demonstrated correlations between radiopharmaceutical uptake intensity and glioblastoma aggression, which, when analyzed alongside MRI features, may enhance diagnostic accuracy and provide deeper insights into glioblastoma biology.^79^ Combining these modalities within a machine learning framework could enable a more holistic understanding of tumor behavior, ultimately improving personalized treatment planning and prognostication.

Radiomic features and metrics can underpin biological processes driving glioblastoma progression. A DTI-based radiomic model identified a high-risk group that significantly correlated with distinct malignant tumor processes, such as DNA damage response, proliferation, and complex cellular functions.^2^ A low-risk group was significantly associated with synapse-related processes.^2^ Radiogenomic studies, based on MRI radiomic models, have revealed significant associations between prognostic radiomic features and key pathways in glioblastoma.^80^ These studies identified links to tumor proliferation pathways, including wingless-related integration site signaling, the P53 pathway, and the PI3K/AKT pathway.^17,81^ In addition, radiomic features were found to correlate with pathways involved in cell differentiation, cell adhesion, and angiogenesis.^16^

### Role of Radiomics in Guiding Therapy

Radiomic features show promise for noninvasive diagnosis and prognosis in glioblastoma, with some models reaching over 90% specificity. However, even high specificity is unlikely to fully replace biopsies due to the tumor's complexity and heterogeneity. Radiomics should complement, not replace, histopathological examination, and be integrated with molecular profiling and other clinical data for a comprehensive strategy. Although radiomics could reduce the biopsy need in specific cases, it remains part of a broader decision-making process. Future advancements may improve specificity, but the focus should be on developing a robust, multimodal approach.

## DISCUSSION

The comprehensive neuroradiological imaging approach outlined in this review highlights the crucial role of advanced imaging in glioblastoma care, from screening to surgical planning and post-treatment surveillance. MRI and DTI are essential for accurately delineating tumor size, location, and involvement of adjacent structures, aiding diagnosis and treatment planning while minimizing brain damage. Radiological features, such as FLAIR signal in the SVZ, and metabolic heterogeneity assessed with labeled radiotracers provide insights into tumor aggressiveness and treatment sensitivity. Imaging biomarkers such as ADC and rCBV help understand tumor heterogeneity and survival outcomes. Radiomics extracts detailed features from neuroimaging for a nuanced tumor characterization, and the integration of machine learning models, especially CNNs, enhances noninvasive diagnosis and molecular characterization, predicting IDH status, genetic mutations, and tumor behavior (Table).

**TABLE.** Neuroimaging Nuances Informing Intertumoral Heterogeneity in Glioblastoma

Imaging modality	Component/finding	Impact	Ref
MRI/DTI	Decreased isotropic diffusion and increased anisotropic diffusion in the nonenhancing tumor subregion	Increased infiltrative environment for targeted therapies	Li et al,^14^ 2019
MRI/DTI	Widespread connectome disruptions	Lower preoperative performance, impaired cognitive function, and worse survival	Wei et al,^48^ 2022
MRI/DTI	Abnormal anisotropic diffusion and contrast-enhancing region	Predicts glioblastoma recurrence and survival	Mayrand et al,^49^ 2022; Simon et al,^50^ 2022
MRI	Lack of cystic component	Increased tumor-associated myeloid cells and tumor-promoting macrophages	Zhou et al,^34^ 2018
MR Spectroscopy	Hyperpolarized carbon 13–labeled metabolites	Permits the assessment of pyruvate metabolism in the tumor	Zaccagna et al,^56^ 2018
MRI	Higher signal intensity in FLAIR, rCBV, and T1 contrast, and lower intensity in ADC and DTI-p	Peritumoral areas that exhibit increased progression areas and invasion	Yan et al,^76^ 2020

ADC, apparent diffusion coefficient; DTI, diffusion tensor imaging; FLAIR, fluid-attenuated inversion recovery; rCBV, relative cerebral blood volume.

Treating glioblastoma based on imaging alone, without a tissue diagnosis, is gaining interest, especially when biopsy risks are high. Advances in MRI-based radiomics and radiogenomics have improved noninvasive tumor characterization. In cases where biopsy is unsafe, imaging-based treatment may be considered. However, glioblastoma's molecular complexity highlights the need for tissue diagnosis in most cases, as it guides personalized therapies that imaging alone cannot fully support. Despite the high specificity of some radiomics models, the risk of misdiagnosis remains, affecting treatment outcomes. Therefore, while imaging-based treatment might be viable in select cases, tissue diagnosis should remain the standard whenever possible. A multimodal approach, combining imaging and molecular diagnostics, is recommended for now.

Despite notable advancements in neuroimaging and machine learning, challenges persist in their routine clinical use for glioblastoma management.^82^ Critical among these is the necessity for large, representative, and cost-effective data sets.^22^ Future research should prioritize efficiency, diverse data sets, and the development of practical clinical decision support systems. Optimizing machine learning models for classification with smaller sample sizes is crucial, and collaboration for data sharing among institutions is essential to compete with private-sector entities. Moreover, acknowledging that machine learning MRI processing approaches are often presented in conference proceedings is vital for staying updated on field advancements.^22^ Standardization of imaging protocols and structured reporting is imperative to address the variability in AI algorithm performance across diverse health care networks. Establishing ground-truth genomics for CNNs and MRI, particularly in glioblastoma, presents additional challenges, necessitating potential changes in clinical practice. Seamless integration of CNNs into routine workflow is paramount to avoid disruption, requiring a deeper understanding of CNN decision-making processes to gain full acceptance from neuroradiologists, neuropathologists, and neurosurgeons.^82^

Several near-term projects could significantly affect glioblastoma diagnosis and management. First, creating a multimodal imaging biomarker panel combining MRI radiomics, PET, and DTI could noninvasively identify glioblastoma molecular subtypes, potentially reducing biopsy need. Second, enhancing machine learning algorithms to predict treatment response from preoperative imaging could personalize treatment plans, improving outcomes. Third, radiogenomic mapping of recurrence patterns could detect early signs of recurrence, enabling timely interventions. Last, a prospective study using serial imaging and radiomics to monitor tumor evolution could allow for real-time therapy adjustments. These initiatives could advance noninvasive, precision management of glioblastoma.

## CONCLUSION

Neuroimaging and machine learning are powerful tools for studying glioblastoma heterogeneity. They improve tumor understanding, aiding personalized clinical management. This synergy promises better outcomes for patients, highlighting radiomics' role in survival prediction and clinical application. Although imaging biomarkers, including those derived from advanced radiomics and radiogenomics, have shown potential for independent prognostication and guiding therapy, they are not yet fully validated as replacements for biopsy-based genomic and proteomic analyses. These biomarkers should be viewed as complementary to molecular diagnostics, which remain essential for a comprehensive understanding of tumor biology and for the personalization of treatment strategies in glioblastoma. As imaging techniques and models continue to improve, their role in clinical practice will likely expand, but for now, they should be integrated within a broader, multimodal framework. This emphasizes the need to continue to include emerging imaging techniques, refine machine learning models, and gain a deeper understanding of the intricate interplay between imaging features and underlying molecular and cellular processes.

## COMMENTS

This is a very interesting and timely review article on the application of machine learning–based radiomics in the diagnosis of glioblastoma. This paper leverages the data gleaned from MRI and DTI ie, radiomics to train machine learning models, in particular machine learning techniques, such as Convolutional Neural Networks (CNNs). This review explores how recent advances in neuroimaging and machine learning enabled refined classification, identification of invasive tendencies, and prediction of patient survival in patients with glioblastoma. I agree with the authors in that while significant progress in this field has been made, further innovation is necessary to address remaining challenges and knowledge gaps and further refine these methodologies. Future studies including combining MRI radiomics, PET, and DTI could noninvasively identify GBM molecular subtypes and may in the future potentially reduce the need for biopsy in certain cases. Machine learning algorithms show promise in differentiating tumor progression from pseudo progression, which would be of great clinical utility. The authors emphasize that radiomics are still mainly research-based and currently not widely in clinical use and though models have shown promising specificity and sensitivity they should complement not replace biopsy. This paper should act as primer for those looking to develop similar studies of their own.

**Cormac G. Gavin**

*London, UK*
